# Multiscale X-ray imaging using ptychography

**DOI:** 10.1107/S1600577518007221

**Published:** 2018-06-17

**Authors:** Simone Sala, Venkata S. C. Kuppili, Stefanos Chalkidis, Darren J. Batey, Xiaowen Shi, Christoph Rau, Pierre Thibault

**Affiliations:** a Department of Physics and Astronomy, University College London, London WC1E 6BT, UK; b Diamond Light Source, Harwell Science and Innovation Campus, Didcot OX11 0DE, UK; c Department of Physics and Astronomy, University of Southampton, Southampton SO17 1BJ, UK

**Keywords:** ptychography, X-ray imaging, coherent diffraction, phase contrast, scanning transmission X-ray microscopy

## Abstract

The success of ptychography and other imaging experiments at third-generation X-ray sources is apparent from their increasingly widespread application and the improving quality of the images they produce both for resolution and contrast and in terms of relaxation of experimental constraints. The wider availability of highly coherent X-rays stimulates the development of several complementary techniques which have seen limited mutual integration in recent years. This paper presents a framework in which some of the established imaging techniques – with particular regard for ptychography – are flexibly applied to tackle the variable requirements occurring at typical synchrotron experiments. In such a framework one can obtain low-resolution images of whole samples and smoothly zoom in on specific regions of interest as they are revealed by switching to a higher-resolution imaging mode. The techniques involved range from full-field microscopy, to reach the widest fields of view (>mm), to ptychography, to achieve the highest resolution (<100 nm), and have been implemented at the I13 Coherence Branchline at Diamond Light Source.

## Abstract 2

A detailed description of an imaging protocol which integrates both near- and far-field ptychography with full-field and scanning transmission X-ray microscopy in multiscale imaging experiments at synchrotron endstations is given.

## 1. Introduction   

X-ray ptychography was first conducted successfully in the mid-1990s (Chapman, 1996 ▸, 1997 ▸). It caught the interest of the X-ray imaging community a decade later, with the demonstration of efficient iterative reconstruction algorithms (Rodenburg *et al.*, 2007 ▸; Thibault *et al.*, 2009 ▸). It is now one of the most actively developed synchrotron-based techniques, to the benefit of its applicability and achievable resolution (Schropp *et al.*, 2012 ▸; Holler *et al.*, 2014 ▸; Guizar-Sicairos *et al.*, 2015*a* ▸). At coherent X-ray beamlines, ptychography routinely delivers absorption and phase-contrast images of extended two-dimensional and three-dimensional specimens with a few tens of nanometres resolution. Already established within the far-field propagation regime, ptychography has also been demonstrated successfully in the near-field regime, further widening its range of applicability (Stockmar *et al.*, 2013 ▸).

However, being scanning techniques, both far- and near-field ptychography impose an obvious trade-off between the size of the imaged area and the time necessary to scan the X-ray beam over it. It therefore becomes a key asset to be able to rapidly identify specific areas of interest on which to apply lengthy high-resolution scans. A common approach to tackle this issue is the use of an X-ray camera or X-ray eye, *i.e.* a video camera coupled with a scintillator which provides live feedback on sample position and orientation. Similarly, an online visible-light microscope is sometimes used for the same task. Scanning transmission X-ray microscopy (STXM) too makes a well known and strong candidate able to retrieve coarse wide images. This latter technique has been developed at full-field X-ray microscopy beamlines (Chapman *et al.*, 1995 ▸; Gianoncelli *et al.*, 2006 ▸; Stampanoni *et al.*, 2010 ▸; Kaulich *et al.*, 2011 ▸) providing an efficient way of probing even wide areas with little processing requirements and with an achievable resolution determined by the used beam size. When exploiting tightly focused beams, STXM can produce high-quality images resolving nanometric features (Mohacsi *et al.*, 2017 ▸). However, it has been shown that STXM scans can also support far-field ptychography experiments by exploiting the same geometry and requiring significantly less processing to provide wide overviews of whole specimens (Thibault *et al.*, 2008 ▸; Shapiro *et al.*, 2014 ▸; da Silva *et al.*, 2015 ▸; Guizar-Sicairos *et al.*, 2015*b* ▸).

Stemming from this application, we implemented and established a flexible protocol for both near- and far-field ptychography experiments to be performed at an X-ray beamline in combination with other imaging methods to obtain both wide field-of-view images of whole specimens and submicrometre-resolution images of specific regions of interest. For the far-field propagation regime this is achieved by combining ptychography with STXM while in the near-field an approach which exploits full-field transmission images produced by a wide parallel beam is used instead. For simplicity, this latter approach will be referred to as parallel-beam holography throughout this article.

In the *Methods* [Sec sec2] section we present the geometrical configurations and relative analysis methods used within the proposed protocol as well as their working details for the specific beamline at which they have been first implemented and established. A discussion follows comparing the techniques available within the protocol and the extent to which they complement each other. We then show some experimental results obtained through the application of different combinations of those techniques to illustrate the wide range of length scales they can access.

## 2. Methods   

The protocol developed for the experiments discussed in the present article includes three different geometries accessible at the I13-1 Coherence Branchline at Diamond Light Source (Rau *et al.*, 2011 ▸; Kuppili *et al.*, 2017 ▸) which can be remotely and reversibly swapped *via* simple motor translations. This approach allows for four different and complementary propagation-based scanning X-ray microscopy techniques to be used interchangeably thus providing a flexible means to investigate extended samples at multiple scales. These techniques are parallel-beam holography (PBH), scanning transmission X-ray microscopy (STXM) and both near-field (NFP) and far-field ptychography (FFP). While PBH returns plain unprocessed transmission images, STXM is able to produce differential phase-contrast and dark-field images too when a suitable detector is used, *i.e.* a position-sensitive detector such as a segmented or pixelated one. Both NFP and FFP lead to the reconstruction of the complex-valued transmission function of the projection of the imaged area thus providing both absorption- and phase-contrast images.

**Figure 1:**
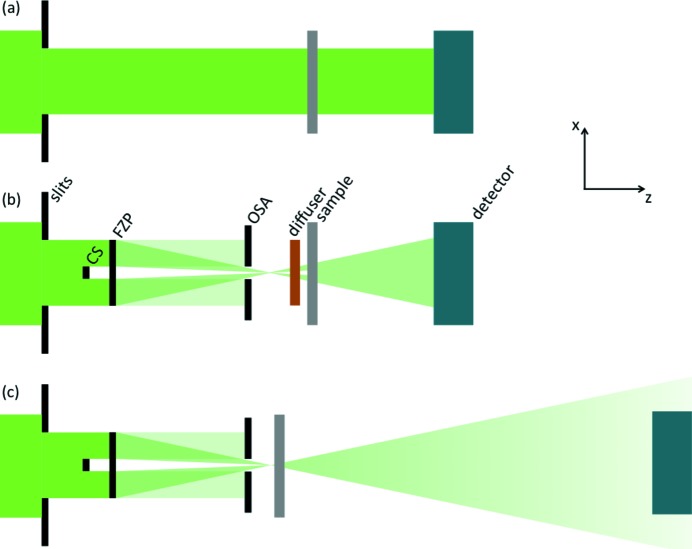
Schematic representation of the three geometries used within the proposed protocol. The elements annotated on the figure are beam-shaping slits, a central beam stop (CS), a Fresnel focusing zone plate (FZP), an order-sorting aperture (OSA), a cardboard diffuser, the sample and a detector. (*a*) Geometry suited for parallel-beam holography, generating wide field-of-view and low-resolution images. (*b*) Geometry suited for near-field ptychography, producing intermediate to small field-of-view and sub-micrometre-resolution images. (*c*) Geometry suited for scanning transmission X-ray microscopy producing images within a wide range of fields of view and resolutions and suited for far-field ptychography for small field-of-view and high-resolution images.

The used geometries are represented in Fig. 1 ▸ and have been applied for measurements with hard X-rays (9.1–9.7 keV). They involve different combinations of the same components: moving downstream along the optical axis (*z*) a pseudo-channel-cut crystals Si monochromator and some beam-shaping adjustable slits which determine the secondary source size are used throughout each experiment. In some configurations further optical elements used are a 40 µm central beam stop (CS), a 400 µm Fresnel focusing zone plate (FZP) with 150 nm outermost zone width, a 25 µm order-sorting aperture (OSA) and a cardboard diffuser, each individually mounted on three-axis translation stages. The specimen is mounted on top of a high-precision translation and rotation stage. Finally, detectors are positioned further downstream with no other optical element needed between these and the specimen as only propagation-based microscopy techniques are involved. Two different detectors can be used interchangeably thanks to additional translation stages. Since near-field imaging requires a small pixel size, measurements in this regime are collected with a scintillator-coupled pco.edge 5.5 cSMOS camera. The detector has 2560 × 2160 pixels, 6.5 µm each, giving a magnified pixel size of 0.29 µm when the scintillator is coupled through a 20× objective lens. Far-field measurements are made with EXCALIBUR, a photon-counting detector based on the Medipix3 chip with 2069 × 1796 pixels, 55 µm each (Marchal *et al.*, 2013 ▸).

In the first geometry (Fig. 1*a* ▸), the parallel beam shaped by the adjustable slits illuminates the specimen directly with no further optical element involved and is detected by the scintillator camera positioned in the near-field propagation regime. This way holograms of areas of the sample up to a few millimetres across can be measured. For this experiment, holograms have been collected by scanning samples on a rectangular grid transverse to the beam with a 250 µm step size, and have then been stitched together to form larger absorption-contrast images typically containing the whole sample. Each of these stitched holograms took a few minutes of measuring time, the widest one requiring just under 20 min to be collected and spanning an area 8 mm wide for a total of 372 megapixels.

The second geometry (Fig. 1*b* ▸) is used to run NFP scans and is achieved by inserting the focusing optics (CS, FZP, OSA) and the diffuser into the beam path. The focusing optics needs careful alignment aimed at having mostly the first-order beam diffracted by the FZP reaching its focal distance whereas most of the lower and higher orders are shadowed out by the CS and OSA. This alignment only needs to be carried out or tested once at the beginning of each experiment: in fact, unless large changes in beam energy are foreseen, the properties of the focus remain untouched throughout. The sample is then positioned downstream of the focus such that it is illuminated by a 40–80 µm perturbed divergent beam which then propagates in the near-field to the scintillator camera. Such a geometry allows complex-valued images with sub-micrometre resolution to be produced from ptychographic scans run on relatively wide areas (40–100 µm) typically taking under 2 min to be collected.

The third geometry (Fig. 1*c* ▸), suited for both STXM and FFP measurements, is produced from the second by swapping the scintillator camera with the photon-counting detector, removing the diffuser, and translating the sample closer to the focal position. Like the wide holograms obtained from the first geometry, STXM images of whole samples of several tens of micrometres can easily be generated in this geometry. Using this technique, the acquisition time increases with the inverse square of the beam size on the sample plane while the resolving power increases linearly: this leads to a trade-off that needs to be carefully balanced depending on the experimental requirements and which is further commented upon in Appendix *B* [App appb]. For example, for this experiment a 144 µm × 144 µm STXM image with 8 µm pixel size was collected in just under 7 min. On the other hand, ptychographic reconstruction of diffraction patterns collected in this geometry leads to the achievement of the highest half-period resolution, here about 35 nm, with scanning times usually of the order of a few minutes, but reaching more than 10 min for the largest detector exposure times and fields of view.

All these geometries are compatible with three-dimensional imaging experiments as the presence of a rotation stage beneath the sample stage allows conventional tomography to be carried out in any of the configurations. This approach has in fact been already established for both FFP (Dierolf *et al.*, 2010 ▸) and NFP (Stockmar *et al.*, 2015 ▸) where two-dimensional projections are reconstructed at several sample orientations thus forming a tomographic dataset suited for the retrieval of quantitative phase-contrast volumetric information. Furthermore, these geometries also allow for on-the-fly scans to be performed, which can be handled by ptychographic reconstruction algorithms and are expected to greatly decrease the acquisition time for each scan – even by more than one order of magnitude – which is a critical factor for most three-dimensional measurements (Pelz *et al.*, 2014 ▸; Deng *et al.*, 2015 ▸). Finally it is worth noting that for the experiment reported here all specimens satisfy the projection approximation. It is expected that extending these methods to samples significantly thicker than the depth of field would require taking into account the probe changes as it travels within the sample. This is relevant in particular for the high-resolution techniques, namely both near- and far-field ptychography, and some algorithmic approaches have been successfully proposed to tackle this issue (Maiden *et al.*, 2012 ▸; Suzuki *et al.*, 2014 ▸; Tsai *et al.*, 2016 ▸).

Beyond the experimental setup, the availability of powerful data analysis tools is essential to fully benefit from the flexibility of multiscale experiments. There already exist some established packages for ptychography (Maiden & Rodenburg, 2009 ▸; Marchesini *et al.*, 2016 ▸). For this study we used *PtyPy* (Enders & Thibault, 2016 ▸), which offers ptychographic reconstruction algorithms for both far-field and near-field datasets. The reconstructions presented in this article stemmed from customized parameters but were all based on the same principles. A few hundred (500–1000) iterations of the difference map algorithm were followed by several hundred (1500–3000) iterations of maximum-likelihood refinement, using a few (two to five) mixed states to account for various sources of loss of coherence, yielding the sample’s complex-valued transmission function, from which absorption and phase-contrast images can be generated. *PtyPy* also provides a convenient library to handle and process scanning datasets. For PBH, each hologram is fed into a data management container and a wide field-of-view image is stitched together through a procedure consisting of a weighted sum of each flat-normalized frame taking into account the motor positions associated with each one of them. STXM images are also generated using *PtyPy*’s library, which we complemented with a simple frame-by-frame analysis of the collected diffraction patterns: integration leads to transmission images, while computation of each diffraction pattern’s centre of mass leads to refraction images corresponding to differential phase contrast images.

## 3. Results and discussion   

**Figure 2:**
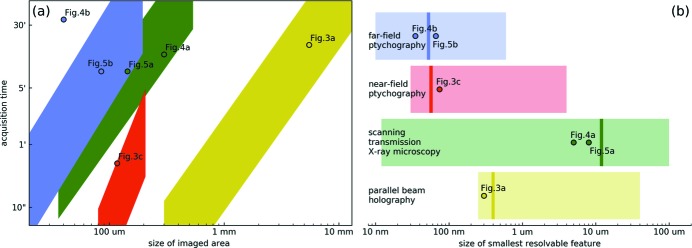
Comparison between imaging techniques available within the proposed protocol. Colour-coded techniques domains in logarithmic space of (*a*) acquisition time and scanning area and (*b*) size of smallest resolvable feature based on typical experimental parameters. In (*b*) transparent domains indicate the regimes accessible in general through each technique and opaque lines indicate the image pixel size obtained with the experimental parameters considered for (*a*). Values relative to experimental results presented in other figures are annotated as circles colour-coded to technique. Colour-coding is labelled in (*b*).

A wide range of length scales can be investigated exploiting the experimental protocol described in the *Methods* [Sec sec2] section. This can be represented as in Fig. 2 ▸ where comparisons between scanning areas, acquisition times and resolving powers best accessible with each technique within the proposed protocol are schematically summarized highlighting their complementarity. Fig. 2(*a*) ▸ shows the domain of each colour-coded technique in the logarithmic space of acquisition time and scanning area where the former represents the time required to complete a whole scan and the latter the size of the image retrieved through such scan. For simplicity, a one-dimensional value has been chosen to represent the scanning area which is assumed to be square. All domains refer to geometries and setup elements selected to be representative of typical experimental conditions – as described more extensively in Appendix *A* [App appa] – and closely resembling those presented in the *Methods* [Sec sec2] section. On the other hand, Fig. 2(*b*) ▸ shows a comparison of the different length scale regimes accessible with each technique. The transparent colour-coded domain represents the whole range of achievable resolving powers from the smallest resolvable feature found in the literature to the largest still relevant to the proposed protocol. The opaque bar is used to annotate the pixel size of images obtained through each technique based on the experimental parameters chosen as for Fig. 2(*a*) ▸. This can be used as a reliable figure of merit for comparison purposes as it only depends on the chosen experimental geometry whereas the actual resolution achieved within any experiment varies significantly with the imaged specimen and only stems from the image pixel size rather than equating to it. Nonetheless, it could be useful to keep in mind the qualitative relationship between image pixel size and achieved resolution within geometries relatively similar to the ones considered to generate Fig. 2 ▸. Using PBH, it should be expected that only features significantly larger than the image pixel size – typically by one order of magnitude – would be resolvable. This is due to the fact that propagation effects cause blurring in the unprocessed near-field images and give rise to fringes. At the same time, edge-enhancement effects make PBH even more effective as a low-resolution technique suited for investigating the general morphology of an uncharacterized specimen. On the other hand, the actual resolution of STXM images corresponds to the beam size used to produce them which could be slightly larger than their pixel size as the latter is given by the scanning step size. Finally, both NFP and FFP often achieve resolutions of only a few pixels but for these techniques especially these largely depend on the imaged specimen and its properties.

Fig. 2 ▸ is not aimed at giving a rigorous picture of the extent of each domain but rather it acts as a guideline in showing what the typical ranges are for the available techniques providing a tool to compare them and assess which to select for each need. These ranges depend in fact on a combination of variables which cannot all be rigorously represented in the same plot and have been chosen to be representative of those that would be optimally used within the proposed protocol assuming typical near-field and far-field detectors as well as focusing optics. These are sample-to-detector distance, beam size at sample position, scanning step size, overhead and detector exposure time.

Furthermore, the general relations 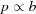 and 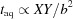 can be found between the image pixel size *p*, the beam size *b*, the imaged area *XY* and the acquisition time 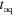, *i.e.* the time required to complete a single two-dimensional scan, which further highlights the trade-offs which need to be taken into account when selecting the most efficient imaging technique for a specific goal.

A further element to be weighed in is the processing time associated with each technique. For well characterized and optimized systems this typically lies in the order of minutes for STXM and PBH which only require limited computational resources. On the other hand, both NFP and FFP involve reconstruction algorithms which run for several iterations before approaching convergence, thus pushing processing time into the hours which is often reduced by employing larger computational resources, namely multiple cores or GPUs.

Taking into consideration the complementarities discussed so far, experiments have been performed at the I13-1 Coherence Branchline at Diamond Light Source exploiting the most efficient combination of techniques suited for each situation. The measurements involved a series of specimens of different sizes and compositions covering the full scale range, the largest sample spanning several millimetres in size and the smallest features lying in the sub-100 nm regime. We report on images from three biogenic specimens representative of extreme cases, *i.e.* located at either end of the accessible length scale range.

The widest sample is a fossil (sample 1), namely a silica slab a few millimetres wide and polished down to a thickness of about 20 µm, extracted from the bone of an extinct fish (genus *Psammolepis*) whose bone repair mechanism is of interest for palaeontologists (Johanson *et al.*, 2013 ▸) and is taken here as an example representative of the high end of the accessible length scale range. Another sample (sample 2) lying at the opposite end is a tricuspid tooth extracted from a limpet (*Patella vulgata*) which has recently been found to feature the highest tensile strength among biological materials (Barber *et al.*, 2015 ▸). Made of 20 nm thin mineral fibres (goethite, α-FeOOH) in an organic matrix [chitin, (C_8_H_13_O_5_N)_*n*_], this sample required access to the highest resolution. Finally, a weakly scattering scale (sample 3) is considered which was extracted from a pansy butterfly (genus *Junonia*) wing. Such scales are known to contain chitin [(C_8_H_13_O_5_N)_*n*_] nano­structures responsible for their colouration thanks to interference effects and which constitute a marker of interest for evolutionary developmental biology (Wasik *et al.*, 2014 ▸). The challenge this sample poses does not lie in the small size of its features but rather in the high degree of accuracy with which these need to be repeatably measured as it is the size of these structures that varies among individuals rather than their general morphology.

**Figure 3:**
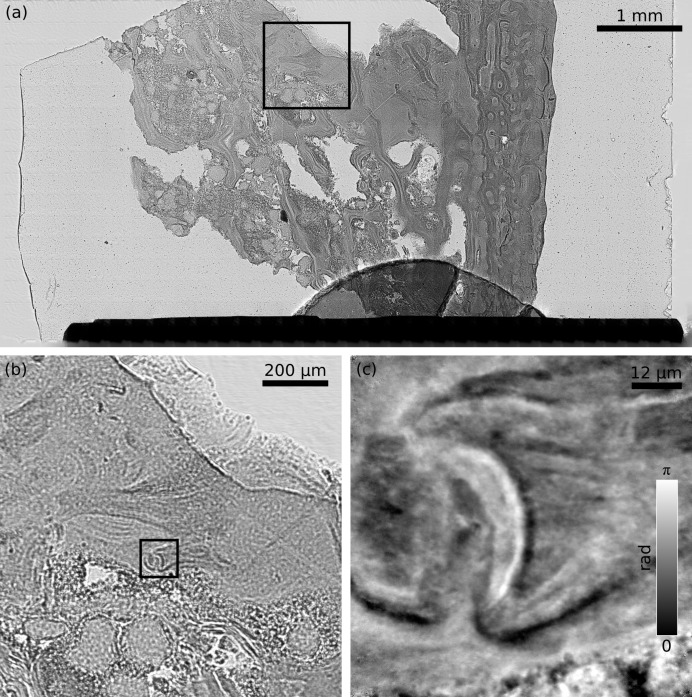
Reconstructions of the fragment of a fossil fish bone (sample 1). (*a*) Transmission image of the whole sample obtained from parallel-beam holography data; arbitrary units. (*b*) Magnified inset from (*a*). (*c*) Relative phase shift from the near-field ptychographic reconstruction of a scan performed on the area of the inset in (*b*).

According to Fig. 2(*a*) ▸ the fastest way to obtain an overview of an area large enough to cover the whole fossil (sample 1) was through a PBH scan. This was performed and returned a low-resolution transmission image (Figs. 3*a*, 3*b* ▸) which made possible the identification of smaller regions of interest. As these were some 80 µm in size and did not require features much smaller than 100 nm to be resolved, they were zoomed into exploiting NFP scans. An example of the images obtained from such scans is shown in Fig. 3(*c*) ▸ as a zoomed-in version of the inset in Fig. 3(*b*) ▸.

**Figure 4:**
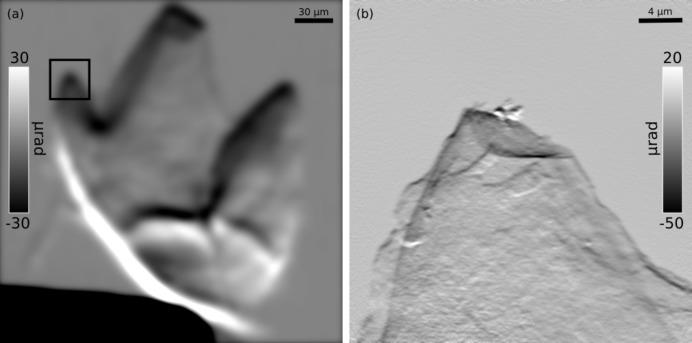
Reconstructions of a limpet tricuspid tooth (sample 2). (*a*) Vertical refraction image of the whole sample obtained from scanning transmission X-ray microscopy data. (*b*) Vertical refraction image of the cuspid from the inset in (*a*) obtained from a far-field ptychographic reconstruction.

**Figure 5:**
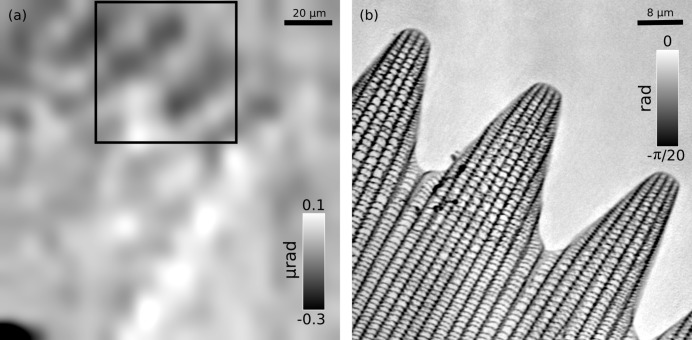
Reconstructions of a butterfly wing scale (sample 3). (*a*) Vertical refraction image of the whole sample obtained from scanning transmission X-ray microscopy data. (*b*) Phase part of the far-field ptychographic reconstruction of a scan performed on the apical region from the inset in (*a*).

A different approach was needed for the other two samples. The tooth (sample 2) was only a few hundred micrometres across and could therefore be imaged whole starting from the far-field geometry directly. Fig. 4(*a*) ▸ shows the refraction image of this sample obtained from STXM data. Despite the low resolution, the general morphology of the sample is apparent and carried enough information to determine on which areas of interest to perform higher-resolution scans: one of these was the tip of one of the cuspids (inset of Fig. 4*a* ▸). As the highest achievable resolving power was necessary to aim at resolving the nanofibres hosted within this sample, a tight FFP scan was performed. The phase part of the reconstruction it produced has been differentiated as in Fig. 4(*b*) ▸ to obtain a refraction image comparable with Fig. 4(*a*) ▸. Some nano­structure can be observed but goethite fibres are not resolved due to the thickness of the specimen. A similar approach was adopted for the butterfly scale (sample 3). This sample stretched just under 200 µm along its longest axis and therefore it too fitted whole within the field of view of a STXM scan which produced the refraction image shown in Fig. 5(*a*) ▸. Higher-resolution FFP scans were run on different regions of the specimen revealing nanometric features. One such scan performed on an apical region clearly highlights the axial ridges and cross-ribs covering the dorsal side of the scale (Fig. 5*b* ▸).

These experimental results can be compared with the general domains in Fig. 2 ▸ where they are annotated as circles coloured according to the technique they exploited. In particular, the values for scanning area and acquisition time for each of the scans used to produce Figs. 3 ▸, 4 ▸ and 5 ▸ are represented in Fig. 2(*a*) ▸ while their pixel size is represented in Fig. 2(*b*) ▸. In the case of Fig. 2(*a*) ▸, some of these annotations lie within the middle-to-upper area of their technique’s domain which is mainly due to the presence of overheads slowing down their acquisition. Also, the FFP scan performed on the tooth (sample 2, Fig. 4*b* ▸) appears as an outlier because of the high degree of overlap and longer exposure times chosen to perform it together with the smaller pixel size aimed at, all factors reducing the accessible field of view per unit time. On the other hand, the image pixel sizes annotated in Fig. 2(*b*) ▸ mostly fall around the expected values generated with the experimental parameters chosen to be representative for each technique (*cf.* Appendix *A* [App appa]). The small discrepancies are simply due to the difference between the geometries and parameters used to perform each of the measurements presented and the general ones considered to produce Fig. 2 ▸.

It can be noted that for all samples a first technique – PBH or STXM – was used as a means to rapidly produce a wide field of view and low-resolution image to be used throughout the experiment to identify specific regions of interest, while a second technique – NFP or FFP – was exploited to obtain higher-quality information on such regions with submicrometric resolution. This time-efficient approach is made possible by the flexibility of the experimental protocol first implemented and demonstrated here.

## 4. Conclusions   

We presented an experimental protocol which can be applied to most setups used for coherent X-ray imaging experiments offering increased flexibility in the selection of several parameters, chiefly the size of the imaged area and the sought resolving power. Any experiment involving wide specimens featuring structures at different scales or designed to retrieve a variety of low- and high-resolution images can benefit from it. We discussed the extension of its applicability and showed some examples of its implementation within experiments for which low-resolution images of whole samples have been produced in order to rapidly identify smaller regions of interest on which to run more time-consuming scans revealing high-resolution features. Given the fact that ptychographic scans are often run at hundreds or even thousands of different sample orientations to produce datasets suited for tomographic analysis, the collection of initial wide-field two-dimensional images is even more crucial to pin down the location and extent of a region of interest making its imaging in 3D more time-efficient. Selecting the most suited technique for each situation together with fine tuning the available parameters also helps in making an optimal use of beam time as well as decreasing dose on the sample, thus preventing potential radiation damage.

We envision the experimental protocol proposed here to be routinely applicable at both soft and hard X-ray beamlines involved in propagation-based imaging experiments both in 2D and 3D.

## Appendix A Parameters for techniques domains

The geometries and setup elements selected to be representative of typical experimental conditions exploited for the realization of Fig. 2 ▸ are as follows. A 10 keV X-ray beam and a 400 µm Fresnel focusing zone plate with 150 nm outermost zone width were assumed. Also a mirror-based focusing system which produced a beam with the same divergence could be employed without altering the outcome. A 2000 × 2000 pixel detector with a pixel size of 0.4 µm after optical magnification has been assumed for near-field and a 500 × 500 pixel detector with 50 µm pixel size for far-field. A minimum detector exposure time of 0.8 s has been assumed for all techniques. The chosen beam size varied among techniques such that for PBH it was 300 µm, for NFP it was 80 µm and for STXM and FFP 12 µm. The minimum degree of overlap between the areas illuminated at adjacent scanning points was set to 0% for PBH and STXM, 71% for NFP and 50% for FFP, implying that for each technique each point was illuminated once, 12 times and four times, respectively; this was an approximation which ignored boundary effects, *i.e.* the scanning points framing the scanned area. Finally, the sample-to-detector distances have been determined such that the divergent beam would illuminate about 50% of the near-field detector area and take up at most 21% of the area sampled in Fourier space by the far-field detector thus ensuring a high degree of oversampling.

## Appendix B Dependencies of parameters for techniques domains

We briskly touch on how the different variables affect each technique’s domain: in particular we consider overhead, exposure time, scanning step size and beam size.

Overhead  is caused by hardware limitations and has no effect other than uniformly slowing down the scanning procedure. It is typically the sum of sample motor overhead and communication overhead. The former occurs whenever the sample is translated by discrete steps in between collection of frames at each point of extended scans as both motor translation and settling take a finite amount of time during which no measurement takes place. The latter includes all non-measuring time necessary for the communication to occur among the various hardware components of the setup, typically between motors, controllers and detector. The increase of acquisition time caused by overhead is sometimes overcome by replacing move–settle–measure scans with continuous or on-the-fly scans which require, however, a more sophisticated analysis.

Exposure time (or dwell time)  is more freely adjustable and is usually chosen on the principle of as short as possible and as long as necessary in that the goal is ultimately to collect raw frames with signal-to-noise ratio high enough to retrieve images of the specimen. For STXM and PBH, low or noisy signal can compromise the quality of the reconstruction, mainly in terms of contrast, while for NFP and FFP it could even prevent reconstruction algorithms from converging altogether, *e.g.* when speckle visibility is compromised.

Scanning step size should be selected to be as large as possible in order to reduce the overall acquisition time. In the case of STXM and PBH this could be as large as the beam size as all frames are processed individually and can be stitched together based solely on the recorded motors positions. However, the stitching procedure would benefit from some degree of overlap between adjacent frames which in the case of holograms could even be used to correct for errors in the recorded motors positions. In the case of NFP and FFP, the scanning step size is a fraction of the beam size as enough overlap between adjacent illuminated areas is necessary for the reconstruction algorithms to tackle the phase problem and hence converge. This parameter can also be represented as the degree of overlap (or redundancy) *r* necessary among illuminated areas, *i.e.* the number of times each area is illuminated throughout a scan.

The beam size *b* too should be selected to be as large as possible to accelerate the scanning procedure, always keeping in mind that an increase in beam size entails an increase in the image pixel size for all techniques but PBH for which the pixel size corresponds to the available near-field detector pixel size. So for PBH the beam size is only limited by the size of the beam available upstream of the optics and the beam-shaping slits can be opened wide as long as a reasonably small secondary source size is preserved.

Combining all the variables introduced, one can seek the general dependency of the total acquisition time  per scan. Assuming a square beam  =  = , one obtains

where  represents the number of frames collected and *X* and *Y* the horizontal and vertical sizes of the area imaged within the scan. As  and  are experiment-dependent and *r* is limited by the technique used, the general relation  mentioned in the *Results and discussion* [Sec sec3] section holds.

Similarly, we show the technique-specific dependencies of the achievable image pixel size *p*. As already mentioned, for PBH this is

with  representing the pixel size of the near-field detector.

For STXM, every frame corresponds to a pixel so that when the smallest degree of overlap is used ( = 1) one finds

once more highlighting the trade-off mentioned in the *Methods* [Sec sec2] section.

For NFP,

with  pixel size the the near-field detector, the magnification *M* caused by the divergent beam, the focus-to-sample distance  and the sample-to-detector distance . The geometry is typically fixed such that a specific area of the near-field detector is illuminated, so the focus-to-detector distance  is constant. As  is a hardware parameter, this leaves , but  is chosen to obtain a specific beam size onto the sample and, in the case of a Fresnel zone plate of diameter  and focal length *f*,  = . Once again treating hardware parameters as constant leads to the general relation

similar to the STXM case.

Finally, for FFP,

with the X-ray beam wavelength λ, the sample-to-detector distance , the far-field detector size *D*, number of pixels *N* and pixel size *p*. Keeping λ and the detector parameters as constant, one obtains  which for speckle-resolution constraints needs to satisfy  >  which once more reduces to

similarly to the STXM and NFP cases.

We thus derived the general dependencies  and  mentioned in the *Results and discussion* [Sec sec3] section, highlighting the relations between selected beam size and image pixel size, size of imaged area and total acquisition time.
